# PRC2 disruption in cerebellar progenitors produces cerebellar hypoplasia and aberrant myoid differentiation without blocking medulloblastoma growth

**DOI:** 10.1186/s40478-023-01508-x

**Published:** 2023-01-12

**Authors:** Abigail H. Cleveland, Daniel Malawsky, Mehal Churiwal, Claudia Rodriguez, Frances Reed, Matthew Schniederjan, Jose E. Velazquez Vega, Ian Davis, Timothy R. Gershon

**Affiliations:** 1 Department of Neurology, University of North Carolina at Chapel Hill, Chapel Hill, NC 27599 USA; 2 Cancer Cell Biology Training Program, University of North Carolina at Chapel Hill, Chapel Hill, NC 27599 USA; 3 Wellcome Sanger Institute, Wellcome Genome Campus, Hinxton, UK; 4 Department of Pathology and Laboratory Medicine, Emory University School of Medicine, Atlanta, GA 30322 USA; 5 Department of Pediatrics, University of North Carolina at Chapel Hill, Chapel Hill, NC 27599 USA; 6 Department of Pediatrics, Emory University School of Medicine, Atlanta, GA 30322 USA; 7 Children’s Center for Neurosciences Research, Emory University School of Medicine, Atlanta, GA 30322 USA; 8 Aflac Cancer and Blood Disorders Center, Emory University School of Medicine, Atlanta, GA 30322 USA

**Keywords:** Medullomyoblastoma, PRC2, H3K27me3, Fate commitment

## Abstract

We show that Polycomb Repressive Complex-2 (PRC2) components EED and EZH2 maintain neural identity in cerebellar granule neuron progenitors (CGNPs) and SHH-driven medulloblastoma, a cancer of CGNPs. Proliferating CGNPs and medulloblastoma cells inherit neural fate commitment through epigenetic mechanisms. The PRC2 is an epigenetic regulator that has been proposed as a therapeutic target in medulloblastoma. To define PRC2 function in cerebellar development and medulloblastoma, we conditionally deleted PRC2 components *Eed* or *Ezh2* in CGNPs and analyzed medulloblastomas induced in *Eed*-deleted and *Ezh2*-deleted CGNPs by expressing *SmoM2*, an oncogenic allele of *Smo*. *Eed* deletion destabilized the PRC2, depleting EED and EZH2 proteins, while *Ezh2* deletion did not deplete EED. *Eed*-deleted cerebella were hypoplastic, with reduced proliferation, increased apoptosis, and inappropriate muscle-like differentiation. *Ezh2*-deleted cerebella showed similar, milder phenotypes, with fewer muscle-like cells and without reduced growth. *Eed*-deleted and *Ezh2*-deleted medulloblastomas both demonstrated myoid differentiation and progressed more rapidly than PRC2-intact controls. The PRC2 thus maintains neural commitment in CGNPs and medulloblastoma, but is not required for SHH medulloblastoma progression. Our data define a role for the PRC2 in preventing inappropriate, non-neural fates during postnatal neurogenesis, and caution that targeting the PRC2 in SHH medulloblastoma may not produce durable therapeutic effects.

**Supplementary Information:**

The online version contains supplementary material available at 10.1186/s40478-023-01508-x.

## Introduction

During brain development, epigenetic inheritance specifies cell identities, directing progenitor cell differentiation along trajectories determined by their lineage. Thus, rhombic lip progenitors give rise to progeny with specific neural fates, including cerebellar granule neurons (CGNs) [1] and unipolar brush cell neurons (UBCs) [2]. Lineage tracing by scRNA-seq shows that CGNPs differentiate into neurons and not into other types of cells [3]. However, hyperactivation of SHH signaling can transform CGNPs, resulting in medulloblastoma [4–7], a primitive neuro-ectodermal tumor that is the most common malignant pediatric brain tumor. While most medulloblastoma cells adhere to a neural fate trajectory [8, 9], a small fraction of tumor cells differentiates as glia [3, 10]. In giving rise to glia, SHH medulloblastoma cells demonstrate increased pluripotency beyond the neural fate trajectory of CGNPs but maintain commitment to typically neuro-ectodermal fates. Epigenetic mechanisms thus maintain neuroectodermal lineage commitment through generations of proliferating progenitors during brain development and through generations of proliferating tumor cells in medulloblastoma.

PRC2 is a chromatin regulatory complex that has been shown to positively or negatively regulate neural progenitor proliferation in different contexts. The core subunits of the PRC2 include EZH1/2, EED, SUZ12, and RbAp46/48. By regulating trimethylation of H3K27 (H3K27me3), PRC2 suppresses CDKN2A, thus increasing neural progenitor proliferation in the hippocampus [11]. However, PRC2 also inhibits SHH-induced transcriptional regulation by depositing H3K27me3 at bivalent chromatin sites in the promoter regions of SHH pathway target genes [12]. As SHH signaling drives CGNP proliferation [13, 14] and medulloblastoma tumorigenesis [7, 15–18], PRC2-mediated repression of SHH target genes suggests the potential to inhibit proliferation in SHH-driven cells. In addition to affecting proliferation, PRC2 regulates hippocampal progenitor differentiation [11], consistent with of its role in fate commitment in diverse developmental contexts, from drosophila larva [19] to mammalian embryonic stem cells [20, 21]. PRC2 may similarly contribute to cerebellar development by regulating CGNP proliferation and differentiation.

As in brain development, prior studies have shown divergent roles for the PRC2 in cancer, positively or negatively regulating tumor growth in different types of tumors [64, 65]. SHH medulloblastomas upregulate EZH2, the catalytic subunit of the PRC2, suggesting a growth-promoting function [12], and EZH2 inhibition has been proposed as a medulloblastoma therapy [22]. A more complex relationship, however, is suggested by the finding that pharmacologically increasing H3K27me3 levels in cultured medulloblastoma cells decreases tumor cell viability [12]. Moreover, prior studies show different effects on tumor growth in murine medulloblastoma models with widespread or mosaic *Eed* deletion [23]. The role of EZH2 in catalyzing inhibitory H3K27me3 marks and its non-canonical PRC2-independent functions has raised questions about the therapeutic potential of EZH2 inhibition [24–26].

To determine how PRC2 components affect cerebellar development, we deleted *Eed* conditionally in the CGNP-specific *Atoh1*. We then compared CGNP proliferation, apoptosis, differentiation, and gene expression in the resulting *Eed*-deleted mice, *Eed*-intact controls, and to mice with *Ezh2*-deleted CGNPs. To determine PRC2 function in SHH medulloblastoma, we bred *Eed*-deleted and *Ezh2*-deleted mouse lines with mice genetically engineered to develop SHH medulloblastoma from CGNPs. Our data show that CGNPs and medulloblastoma cells require PRC2 to maintain neural fate commitment, and that EED is specifically required for cerebellar growth, but neither EED nor EZH2 are required for medulloblastoma progression.

## Results

### EED is required for proper cerebellar growth

**Fig. 1:**
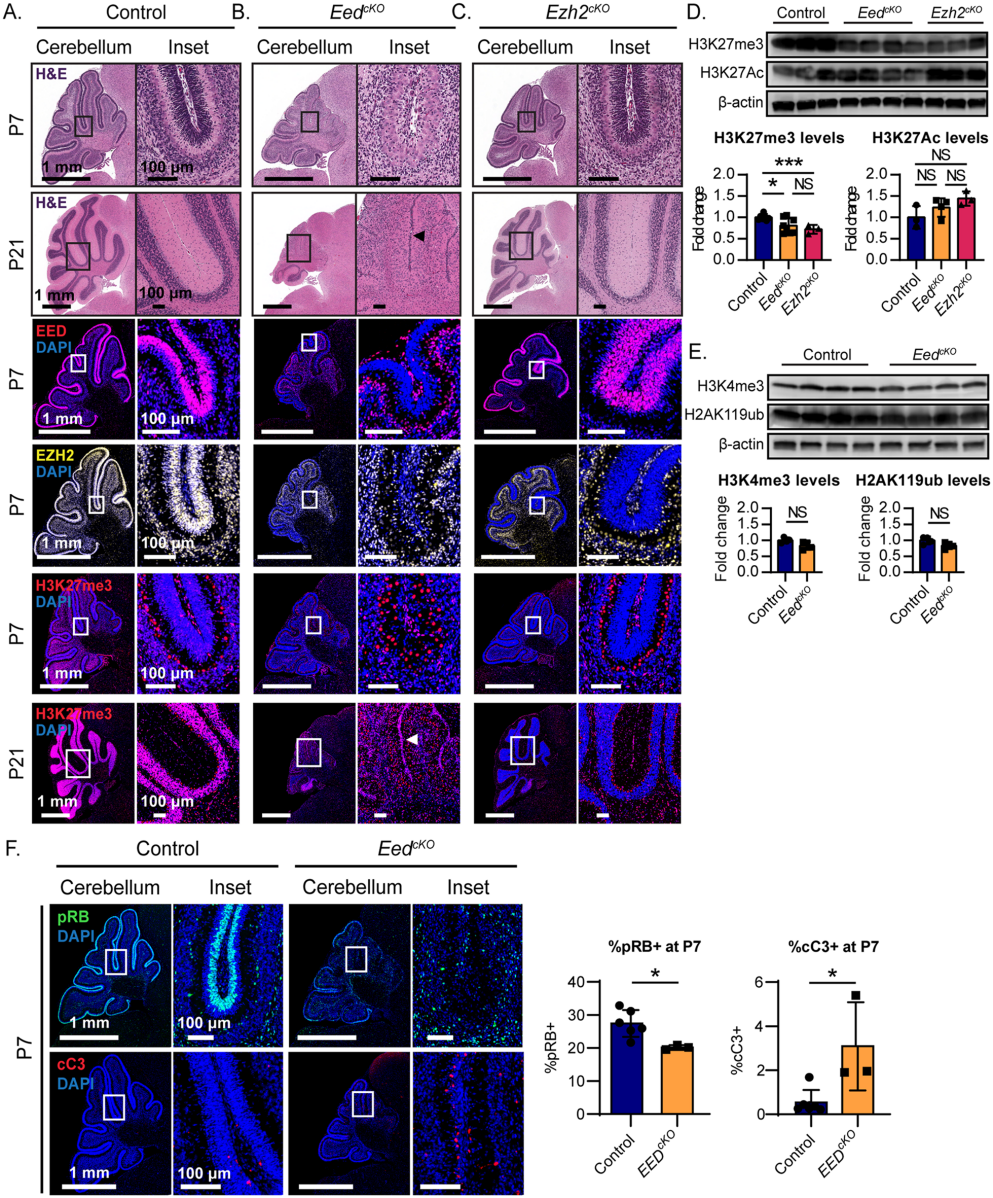
PRC2 function is developmentally regulated in the postnatal cerebellum and is required for normal development. **A**–**C** Representative sagittal sections of **A** control, **B** *Eed*^*cKO*^, and **C** *Ezh2*^*cKO*^ cerebella at the indicated ages. H&E-stained sections show the distribution of cells in the EGL and IGL. IHC shows distribution of EED, EZH2, and H3K27me3, with DAPI counterstain. Red arrowheads highlight Purkinje cells, identified by their typical large nuclei. White arrow indicates a residual EGL population in P21 *Eed* knockouts. **D** Western blot for H3K27me3 and H3K27Ac in replicate samples of indicated genotypes, quantified below, using two-tailed Student’s t-tests. **E** Western blot for H3K4me3 and H2AK119ub in replicate samples of indicated genotypes, quantified below, using two-tailed Student’s t-tests. **F** IHC for pRB and cC3 in representative sections, with quantitative analysis using two-tailed Student’s *t*-tests. *, **, and *** denote *p* < 0.05, *p* < 0.01 and *p* < 0.001 respectively, relative to controls

CGNPs showed robust EED and EZH2 expression during postnatal neurogenesis, with initially low but detectable H3K27 trimethylation that increased as CGNPs differentiated (Fig. 1A). To reduce variation in developmental age when making comparison between genotypes, we used *Eed*^*f/f*^ littermates of *Eed*^*cKO*^ mice to represent normal development. These mice did not inherit Cre and showed intact EED expression. During the period of CGNP proliferation from postnatal day 1 (P1) through P15, cells of the CGNP lineage segregate spatially according to developmental state, with undifferentiated and early differentiating CGNPs in the external granule cell layer (EGL) and differentiated, post-mitotic CGNs in the internal granule cell layer (IGL). At P7, CGNPs throughout the EGL expressed EED and EZH2, and H3K27me3 was detectable in the EGL and IGL (Fig. 1A). H3K27me3 increased in the CGNs of the IGL by P21. Expression of PRC2 components thus continued throughout CGNP development and H3K27me3 correlated with neuronal maturation, suggesting a role for the PRC2 in the differentiation process.

In light these data, we analyzed *Eed* and *Ezh2* function in CGNP using conditional genetic deletion. Prior studies showed that conditional *Ezh2* deletion in the dorsal neural tube at embryonic day E12.5 results in cerebellar hypoplasia, with decreased numbers of both Purkinje cells and CGNPs [27]. However, the loss of Purkinje neurons may indirectly alter CGNPs, which depend on SHH released by Purkinje cells in order to proliferate [28]. To disrupt PRC2 genes specifically in CGNPs, we used conditional deletion by expressing *Cre* from the *Atoh1* (aka *Math1*) promoter, which in the cerebellum is CGNP specific [29]. We interbred *Math1-Cre* transgenic mice that express Cre recombinase in the CGNP population with mice harboring conditional alleles of either *Eed* (*Eed*^*fl/fl*^) or *Ezh2* (*Ezh2*^*fl/fl*^), to generate *Math1-Cre/Eed*^*f/f*^ (*Eed*^*cKO*^) and *Math1-Cre/Ezh2*^*f/f*^ (*Ezh2*^*cKO*^) mice.

*Eed* deletion resulted in marked depletion of the CGNP lineage, with markedly less populous EGL in 5/5 P7 *Eed*^*cKO*^ cerebella compared to controls (Fig. 1B). All *Eed*^*cKO*^ mice subjected to immunohistochemistry (n = 3) showed loss of both EED protein and EZH2 protein throughout the EGL (Fig. 1B), consistent with reports that EED loss destabilizes EZH2 [30]. H3K27me3 expression was absent in the *Eed*^*cKO*^ CGNPs, consistent with PRC2 disruption (Fig. 1B). In contrast, Purkinje cells showed robust EED, EZH2, and H3K27me3; however, these cells, which typically localize at the outer margin of the CGNs, were scattered throughout the depopulated IGL and remained identifiable by their typical morphology with large nuclei and cell bodies (Fig. 1B, red arrows). At P21, 5/5 *Eed*^*cKO*^ cerebella were clearly hypoplastic, with no clear IGL, and with inappropriately persistent, small populations of H3K27me3 + cells in the EGL (Fig. 1B; white arrow). Consistent with cerebellar impairment, *Eed*^*cKO*^ mice showed tremor and ataxia and frequently fell while walking. EED was thus required for PRC2 stability, demonstrated by loss of EZH2 protein in *Eed*^*cKO*^ CGNPs, for H3K27 tri-methylation in CGNPs, and for cerebellar growth and function.

*Ezh2* deletion in contrast did not cause neurologic abnormalities or overt cerebellar hypoplasia (Fig. 1C). P7 *Ezh2*^*cKO*^ CGNPs showed intact EED protein expression despite widespread absence of EZH2 (Fig. 1C). H3K27me3 was clearly reduced in *Eed*^*cKO*^ CGNPs and CGNs compared to controls (Fig. 1C). Thus, while H3K27me3 was altered in both *Eed*^*cKO*^ and *Ezh2*^*cKO*^ mice, *Eed* deletion more effectively disrupted the PRC2 complex, as demonstrated by loss of EZH2 in *Eed*^*cKO*^ cerebella, and more profoundly altered cerebellar growth.

We compared chromatin marks in *Eed*^*cKO*^ and *Ezh2*^*cKO*^ cerebellar lysates, using controls mice that did not inherit the Cre transgene. Western blot analysis showed that both *Eed* and *Ezh2* deletions disrupted PRC2 function and we did not identify differences in chromatin marks. Both deletions reduced H3K27me3 as expected (Fig. 1D). However, residual H3K27me3 from cerebellar cells outside the *Atoh1* lineage that were not subject to conditional deletion produce detectable signal that may have obscured differences. We noted a trend toward increased H3K27 acetylation in both *Eed*^*cKO*^ and *Ezh2*^*cKO*^ (Fig. 1D) that was not statistically significant but would be consistent with the previously observed increased H3K27Ac in H3K27me3-depleted embryonic stem cells [31, 32]. *Eed*^*cKO*^ CGNPs did not show differences in H2AK119 monoubiquitination, indicating that PRC1 function was not altered, and did not show differences in H3K4me3 (Fig. 1E). Within the resolution of our western blot assays, therefore, deletion of *Eed* or *Ezh2* resulted in similar chromatin changes, limited to H3K27 modification.

### *Eed* deletion decreases progenitor proliferation and increases apoptosis

We investigated developmental processes that mediate growth failure in *Eed*^*cKO*^ cerebella. *Eed* deletion was previously shown to decrease proliferation and increased apoptosis in hippocampal progenitors [11]. As both decreased CGNP proliferation and increased CGNP apoptosis can cause cerebellar hypoplasia [13, 33–35], we quantified proliferative CGNPs, identified by expression of phosphorylated-RB (pRB) and apoptotic CGNPs, identified by expression of cleaved Caspase-3 (cC3). We used as controls littermates of *Eed*^*cKO*^ mice that had at least one intact Eed allele, including *Eed*^*f/f*^* mice and Math1-Cre/Eedf*^*/*+^ mice. Similar to hippocampal progenitors, *Eed*^*cKO*^ CGNPs showed decreased proliferation and increased apoptosis (Fig. 1F), implicating both processes in the cerebellar hypoplasia of *Eed*^*cKO*^ mice.

### *Eed* deletion produces discrete patterns of transcriptomic change

To resolve the effects of *Eed-*deletion in individual cells, we subjected *Eed*-deleted and control CGNPs to scRNA-seq analysis. We harvested cerebella from 3 replicate P7 *Eed*^*cKO*^ mice and subjected them to Drop-seq bead-based scRNA-seq preparation [66]. We then sequenced the resulting bar-coded libraries and identified cells by bead-specific bar codes. After QC and filtering, we included 2377 cells for analysis. We compared these cells to previously sequenced data from 6631 cells from 5 replicate P7 WT cerebella. To adjust for differences in sequencing depth in *Eed*^*cKO*^ and WT cells, we down-sampled the WT cells to 46.5% of their original depth to achieve similar sequencing depth between conditions, consistent with best practices [36].

**Fig. 2:**
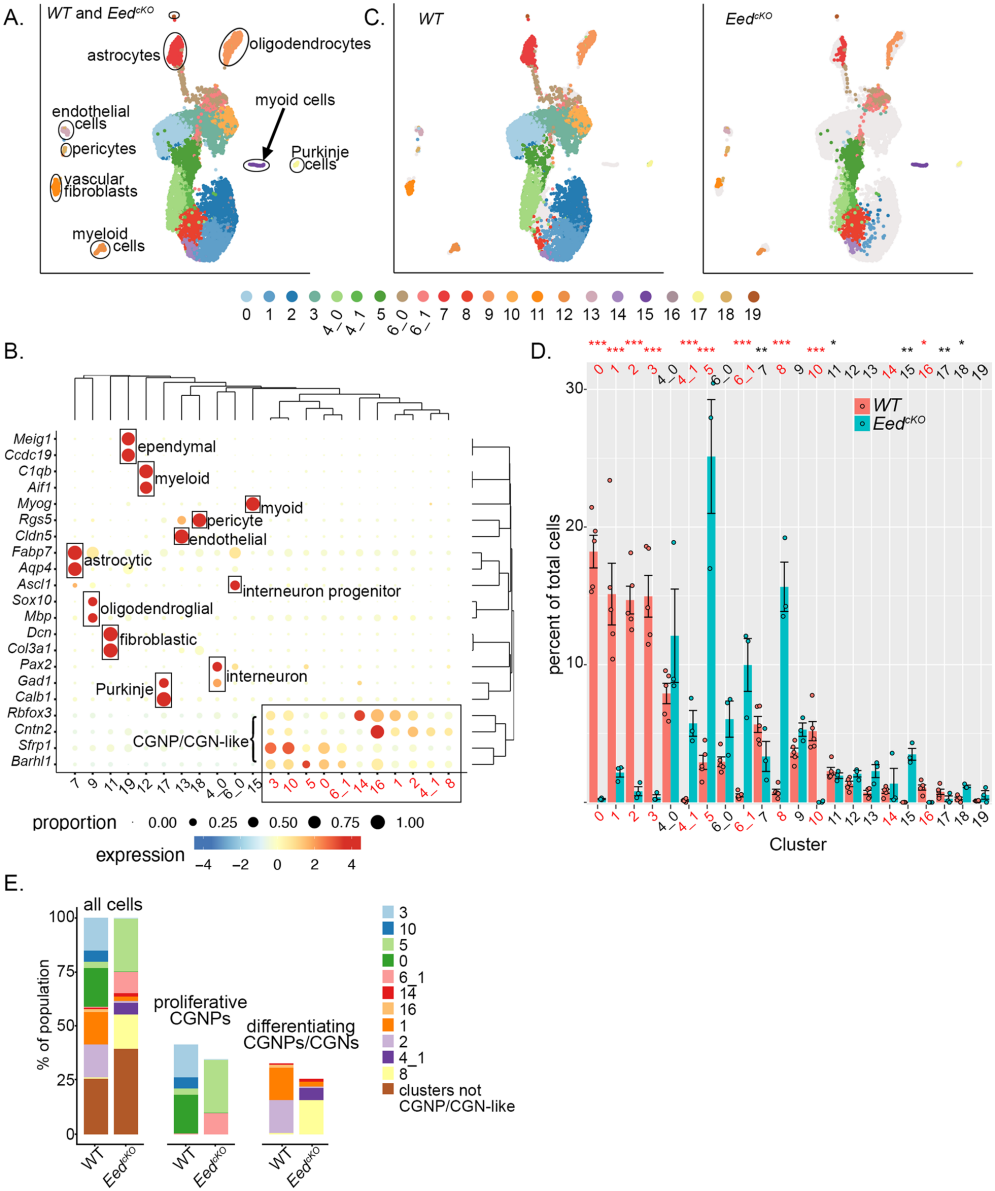
scRNA-seq shows differences in the composition of *Eed*^*cKO*^ cerebella, including a population of myoid cells not found in normal brain. **A** UMAP projection of cells from *Eed*^*cKO*^ and control cerebella, grouped by transcriptomic similarities into color-coded clusters. **B** Bubble Plot shows the magnitude and frequency of the expression of indicated cell type markers in each cluster. The genes and clusters are ordered according to the hierarchical clustering analysis, with groupings indicated along the top and right margins of the Bubble Plot. **C** UMAP projection of cells from *Eed*^*cKO*^ and control cerebella, disaggregated by genotype, with clusters indicated by the same color code as in (**A**). **D** Comparison of each normalized cluster population in *Eed*^*cKO*^ and control cerebella. Dots represent values for individual replicates, bars indicate the means and whiskers indicate the SEM. *** indicates *p* < 0.0001,** indicates *p* < 0.001, * indicates *p* < 0.05; Dirichlet regression was used to compare normalized cluster populations. **E** Bar graphs showing the mean proportional population of each cluster in *Eed*^*cKO*^ and control cerebella, with the subsets of proliferating CGNPs and differentiating CGNPs and CGNs also shown separately for clarity. In **B** and **D**, CGNP and CGN cluster numbers are presented in red for clarity

We subjected scRNA-seq data from *Eed*^*cKO*^ and WT cells to principal component analysis (PCA) and Louvain clustering, as in our prior studies [3, 37, 38], to identify 20 clusters with distinctive gene expression, numbered from 0, most populous, to 19, least populous (Additional file 1: Fig. S1). We then determined cluster-specific gene expression profiles by comparing the expression of each detected gene in cells within the cluster versus all cells outside the cluster (Additional file 2: Data 1). In Clusters 4 and 6, we noted that cluster-specific genes were expressed by discrete subpopulations, suggesting that further sub-clustering of these clusters would be informative. Re-iterative clustering split Cluster 4 into 4_0 and 4_1 and Cluster 6 into 6_0 and 6_1, which showed discrete, cluster-specific patterns of gene expression (Additional file 2: Data 1). We mapped these 22 clusters by color code on the UMAP projection to visualize the different populations (Fig. 2A).

The differential gene expression patterns identified the cell-type of each cluster and demonstrated diverse types of cells that were expected in cerebellar tissue (Fig. 2A,B; Table 1). We identified Clusters 0, 1, 2, 3, 4_1, 5, 6_1, 10, 14, 16, and 17 as CGNP lineage cells in a spectrum of differentiation states, from cycling CGNPs to differentiated CGNs, based on expression of SHH target genes including *Sfrp1*, neural progenitor-related genes including CGNP transcription factor *Barhl1* and axonal pathfinding receptor *Cntn2*, and neuronal genes including the transcription factor *Rbfox3*. Different markers identified diverse types of non-neural cells, including astrocytes (Cluster 7), oligodendrocytes (Cluster 9), vascular fibroblasts (Cluster 11), myeloid cells (Cluster 12), endothelial cells (Cluster 13), pericytes (Cluster 18), and ependymal cells (Cluster 19). Markers also identified neural populations that were outside the *Atoh* lineage but expected in the cerebellum, including gabaergic neural progenitors (Cluster 6_0), identified by expression of *Ascl1* and *Pax3*, gabaergic interneurons (Cluster 4_0) identified by *Pax2* and *Gad1*, and Purkinje cells identified by *Gad1* and *Calb1* [3, 39]. In contrast to all other clusters, which demonstrated markers expected in the brain, Cluster 15 expressed the muscle cell marker *Myog*, suggesting myoid differentiation.

**Table 1:** Cell types identified via scRNA-seq

Cluster #	Designation	*Atoh1*-lineage?	Population difference	*p* value KO versus control
**0**	**Proliferating CGNPs**	**Yes**	**Up in control**	**1.00E−14**
**1**	**Differentiating CGNs**	**Yes**	**Up in control**	**8.40E−14**
**2**	**Early differentiating CGNPs**	**Yes**	**up in control**	**1.00E−14**
**3**	**Late mitotic CGNPs**	**Yes**	**Up in control**	**1.00E−14**
4_0	Interneurons	No		
**4_1**	***Hox* + CGNPs**	**Yes**	**Up in *Eed***^***cKO***^	**1.84E−05**
**5**	**Proliferating CGNPs**	**Yes**	**Up in *Eed***^***cKO***^	**1.03E−07**
6_0	Early interneuron progenitors	No		
**6_1**	***Cdkn2a* + proliferating CGNPs**	**Yes**	**Up in *Eed***^***cKO***^	**3.34E−07**
7	Astrocytes	No	Up in control	0.000213
**8**	***Hox* + differentiating CGNs**	**Yes**	**Up in *Eed***^***cKO***^	**2.91E−10**
9	Oligodendrocytes	No		
**10**	**Mitotic CGNPs**	**Yes**	**Up in control**	**5.67E−10**
11	Vascular fibroblasts	No	Up in control	0.0351
12	Myeloid	No		
13	Endothelial cells	No		
**14**	**CGNs**	**Yes**		
15	Myocytic cells	Yes	Up in *Eed*^*cKO*^	0.000945
**16**	**Early differentiating CGNPs**	**Yes**	**Up in control**	**0.00127**
17	Purkinje cells	No	Up in control	0.0214
18	Pericytes	No	Up in *Eed*^*cKO*^	0.00831
19	Ependymal cells	No		

Cluster numbers and corresponding cell types, with *Atoh1*-lineage cells identified, CGNP and CGN clusters are in bold, and population differences noted between *Eed*^*cKO*^ and WT, with *p* values determined by Dirichlet regression analysis

We disaggregated the UMAP to make separate projections from *Eed*^*cKO*^ and WT mice, demonstrating that each genotype distributed differently across the clusters (Fig. 2C). To compare the cluster populations in *Eed*^*cKO*^ and WT samples statistically, since each replicate contributed different numbers of cells to the analysis, we normalized the population of cells in each cluster from each replicate to the total number of cells from that replicate. These proportional populations were inter-related by the normalization to the whole, and therefore could not be analyzed by individual *t*-tests, which assume independence. We therefore used Dirichelet regression analysis to compare the proportional cluster populations [40].

As suggested by the disaggregated UMAP (Fig. 2C), Dirichelet regression analysis showed that the populations of specific clusters were significantly different in *Eed*^*cKO*^ cerebella compared to controls (Fig. 2D). We found significant differences in the populations of *Atoh1*-lineage cell types that were subject to conditional deletion, and also of clusters outside the *Atoh1* lineage. Within the *Atoh1* lineage, CGNP/CGN Clusters 0, 1, 2, 3, 10, and 16 were more populous in controls, while CGNP/CGN Clusters 4_1, 5, 6_1, and 8 were more populous in the *Eed*^*cKO*^ (Fig. 2D). Outside the *Atoh1* lineage, Purkinje cells, astrocytes, and vascular fibroblasts were increased in controls and pericytes were increased in the *Eed*^*cKO*^. These cell types were not subject to *Eed* deletion, and differences in their populations reflect non-cell autonomous effects. Additionally, the myoid Cluster 15 was present only in the *Eed*^*cKO*^ cerebella (Fig. 2C,D). While proliferative Clusters 5 and 6_1 were relatively increased in *Eed*^*cKO*^ mice, proliferative Clusters 0, 3, and 10 were decreased, resulting in a net decrease in proliferative populations (Fig. 2E), consistent with the decrease in pRB + CGNPs.

### *Eed* deletion permits divergence from the neural fate of CGNPs

**Fig. 3:**
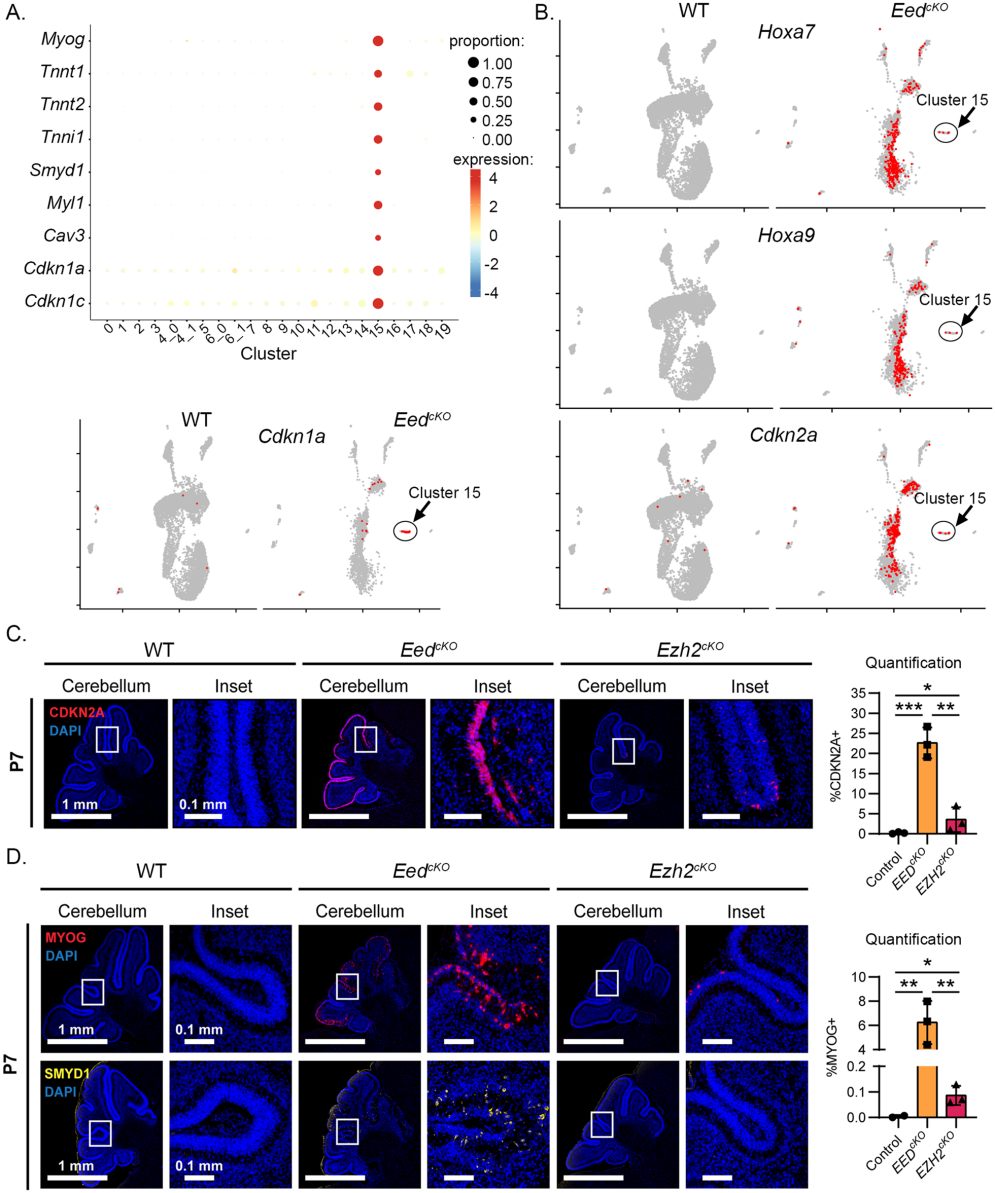
Expression of PRC2 targets in *Eed*^*cKO*^ and *Ezh2*^*cKO*^ cerebella. **A** Bubble Plot shows the magnitude and frequency of the expression of indicated genes in each cluster, and feature plot of *Cdkn1a* + cells (red), color-coded over the UMAP from (2C). **B** Feature plots of *Hoxa9*^+^, *Hoxa7*^+^, and *Cdkn2a*^+^ cells (red), color-coded over the UMAP from (2C). **C** and **D** Representative immunofluorescence in sagittal sections of WT, *Eed*^*cKO*^, and *Ezh2*^*cKO*^ cerebella, showing expression of **C** CDKN2A or **D** MYOG and SMYD1, with DAPI counterstain. The CDKN2A + and MYOG + fractions in cerebellar sections from 3 replicate mice of indicated genotypes are graphed on the right. *Eed*^*cKO*^, and *Ezh2*^*cKO*^ replicates were compared to controls using one-tailed Student’s t-tests and to each other using two-tailed Student’s *t*-tests. *, **, and *** denote *p* < 0.05, *p* < 0.01 and *p* < 0.001 respectively

The *Myog*-expressing Cluster 15 showed multiple genes typical of muscle cells, including troponins, *Myl1*, *Cav3*, and *Smyd1* (Fig. 3A). We did not observe this myoid transcriptomic pattern in any cells from control mice. Cluster 15 cells also up-regulated *Cdkn1a* and *Cdkn1c* (Fig. 3A,B), which are known to be suppressed by the PRC2 in other types of cells [41–44]. Based on the up-regulation of PRC2-suppressed genes, we infer that Cluster 15 cells were within the *Atoh1* lineage, and that conditional *Eed* deletion caused these *Atoh1*-lineage cells to diverge from the expected CGNP trajectory.

### *Eed* deletion permits expression of PRC2-inhibited CDK inhibitors and *Hox* genes

While only Cluster 15 cells showed *Cdkn1a* and *Cdkn1c*, the broad set of *Atoh1*-lineage cells, including CGNPs, CGNs, and Cluster 15 cells expressed other genes typically suppressed by the PRC2, including *Cdkn2a, Hoxa9*, and *Hoxa7* [44–47] (Fig. 3B). Indeed, these markers differentiated the CGNP/CGN clusters enriched in *Eed*^*cKO*^ cerebella (Clusters 4_1, 5, 6_1, and 8) from the CGNP/CGN clusters enriched in WT samples (Clusters 0, 1, 2, 3, 10, and 16). The absence of *Cdkn2a, Hoxa9*, and *Hoxa7* in WT cells (Fig. 3B) indicates that each of these genes are normally suppressed by the PRC2 in CGNP-lineage cells.

### *Ezh2* deletion, like *Eed* deletion, permits myoid differentiation

We used immunohistochemistry (IHC) to determine if specific transcripts from PRC2-target genes were translated in *Eed*^*cKO*^ cerebella, and to probe *Ezh2*^*cKO*^ cerebella for similar patterns of expression. Cells expressing CDKN2A, MYOG, and SMYD1 were absent in controls, including WT cerebella and cerebella from *Eed*^*f/f*^ and *Eed*^*f/*+^ littermates of *Eed*^*cKO*^ mice. As the frequency of CDKN2A + , MYOG + , and SMYD1 + cells in mutant cerebella could not be lower than in control cerebella, we used one-tailed statistical tests to compare mutant to control replicates, and two-tailed statistical tests for comparison between *Eed*^*cKO*^ and *Ezh2*^*cKO*^ genotypes.

*Eed*^*cKO*^ cerebella showed CDKN2A + CGNPs throughout the EGL; the fraction of CDKN2A + cells was significantly greater than in WT or *Ezh2*^*cKO*^ cerebella (Fig. 3C). *Ezh2*^*cKO*^ cerebella showed significantly more CDKN2A + CGNPs than controls, but fewer than *Eed*^*cKO*^ cerebella. Both PRC2 component mutations thus resulted in up-regulation of CDKN2A, with *Eed*^*cKO*^ showing a higher fraction of affected cells.

Both *Eed*^*ckO*^ and *Ezh2*^*ckO*^ cerebella showed cells expressing the muscle cell transcription factor MYOG, while no cells expressed MYOG control cerebella, *Eed*^*cKO*^ cerebella (Fig. 3D). MYOG + cells were significantly more numerous in *Eed*^*ckO*^ than *Ezh2*^*cKO*^ cerebella (Fig. 3D). *Eed*^*ckO*^ cerebella also showed cells expressing the muscle cell chromatin regulator SMYD1, which was absent in *Ezh2*^*ckO*^ and control cerebella (Fig. 3D). Pairwise comparison of SMYD1 expression in *Eed*^*ckO*^ cerebella (present in 3/3) versus *Ezh2*^*ckO*^ (absent in 3/3) or controls (absent in 3/3) using the Barnard’s exact test showed p values of 0.03. Transcripts that were not found in control CGNPs, including *Cdkn2a* and *Myog*, were thus translated into proteins in both *Eed*^*ckO*^ and *Ezh2*^*ckO*^ cerebella, with SMYD1 expression in *Eed*^*cKO*^ cerebella demonstrating an additional myoid gene ectopically expressed.

### Increased activation of the intrinsic apoptotic pathway in ***Eed***^***cKO***^ CGNPs

**Fig. 4:**
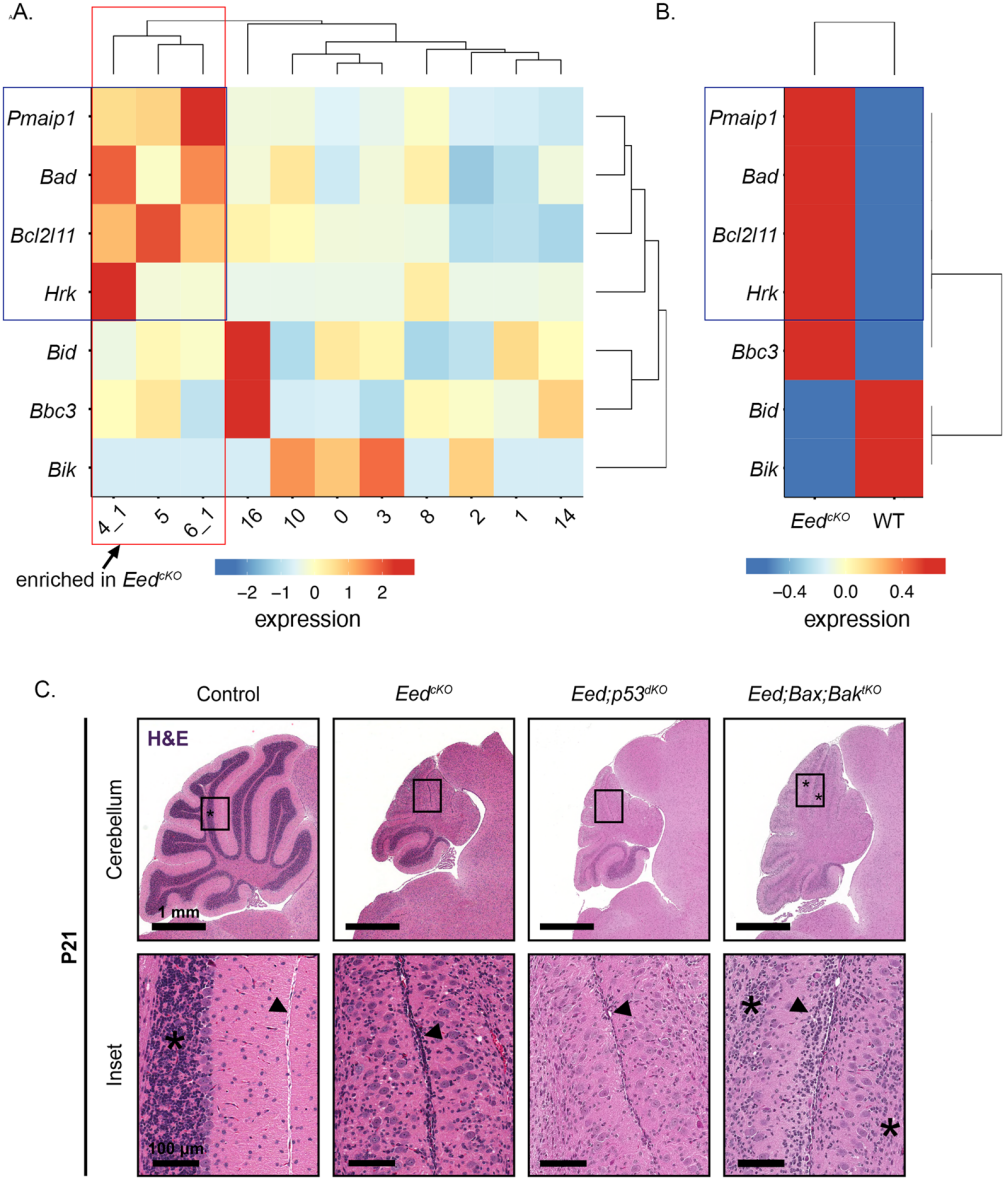
Increased spontaneous, p53-indepenedent CGNP apoptosis contributes to growth failure in *Eed*-deleted cerebella. **A** Heat map of BH3-only gene expression in each CGNP/CGN cluster. Hierarchical clustering based on BH3-only genes grouped together the CGNP clusters that were enriched in *Eed*^*cKO*^ mice (red box), and identified a set of apoptosis regulators (blue box) that was up-regulated in this group. **B** Heat map of BH3-only gene expression in the combined set of CGNP/CGN clusters, comparing *Eed*^*cKO*^ and control genotypes, with the blue box highlighting the same apoptosis regulators as in (**A**). **C** Representative H&E-stained sagittal sections of P21 WT, *Eed*^*cKO*^, *Eed/Trp53*^*dKO*^, and *Eed/Bax/Bak*^*tKO*^ mice. * indicates regions of IGL. Arrow indicates regions of persistent or absent EGL

To probe the mechanisms of increased cell death in *Eed*^*cKO*^ CGNPs, we analyzed p53 function and the expression of genes regulating apoptosis. CGNPs are highly sensitive to p53-mediated activation of the intrinsic apoptotic pathway [48, 49] and also to direct activation the intrinsic apoptotic pathway triggered by changes in apoptotic regulators [35, 50]. *Cdkn1a* expression in Cluster 15 suggested that *Eed* deletion might activate p53-dependent transcription, potentially mediating the observed increased apoptosis. To determine if other apoptotic regulators were altered in *Eed*^*cKO*^ CGNPs, we compared the expression of pro-apoptotic BH3-only genes in the CGNP and CGN clusters of *Eed*^*cKO*^ and control mice (Fig. 4A).

Hierarchical clustering based on BH3-only gene expression distinguished the 3 proliferative clusters enriched in *Eed*^*cKO*^ cerebella, Clusters 4_1, 5, and 6_1, which showed increased expression of *Pmaip1* (aka *Noxa*), *Bad*, *Bcl2l11* (aka *Bim*), and *Hrk* (Fig. 4A). Similarly, comparing the combined set of all cells in CGNP and CGN clusters, *Eed*^*cKO*^ cells showed a distinctive pattern of BH3-only gene expression, with increased *Bbc3* (aka *Puma*), *Pmaip1* (aka *Noxa*), *Bad*, *Bcl2l11* (aka *Bim*), and *Hrk* (Fig. 4B). These data suggested that *Eed* deletion may increase apoptosis by direct activation of the intrinsic apoptotic pathway.

To determine whether p53 signaling or the intrinsic apoptotic pathway contributed to cerebellar hypoplasia in *Eed*^*cKO*^ mice, we combined *Eed* deletion with deletion of either *Trp53* or both *Bax* and *Bak*. We bred *Eed*^*cKO*^ mice with *Trp53*^*fl/fl*^ mice to generate *Math1-Cre/Eed*^*fl/fl*^*/Trp53*^*fl/fl*^ (*Eed/Trp53*^*dKO*^) mice and bred *Eed*^*cKO*^ mice with *Bax*^*fl/fl*^*/Bak*^−/−^ mice to generate *Math1-Cre/Eed*^*fl/fl*^*/Bax*^*fl/fl*^*/Bak*^−/−^ (*Eed/Bax/Bak*^*tKO*^) mice. *Eed/Trp53*^*dKO*^ mice showed cerebellar hypoplasia similar to *Eed*^*cKO*^ mice (Fig. 4C). In contrast, cerebella in *Eed/Bax/Bak*^*tKO*^ mice were markedly less abnormal, with relatively increased CGNs within the IGL and more appropriate layering of Purkinje cells between the IGL and molecular layers (Fig. 4C). *Bax*/*Bak* co-deletion did not fully rescue the effects of *Eed* knockout, as the IGL remained less densely populated than WT controls and the molecular layer contained ectopic cells (Fig. 4C). The absence of rescue in *Eed/Trp53*^*dKO*^ mice indicates that growth failure in the *Eed*^*cKO*^ cerebella was p53-independent. The partial rescue by co-deletion of *Bax* and *Bak*, however, demonstrates that p53-independent activation of the intrinsic apoptosis pathway contributed to cerebellar hypoplasia in *Eed*^*cKO*^ mice. *Bax*/*Bak* co-deletion also increased the myoid population (Additional file 1: Fig. S2), indicating that myoid cells were typically removed from *Eed*^*cKO*^ cerebella by apoptosis.

### PRC2 function is not required for SHH medulloblastoma tumorigenesis

Mutations that disrupt cerebellar growth may identify genes required for growth of medulloblastoma [51] and the PRC2 has been proposed as a target for medulloblastoma therapy. Therefore, to determine whether medulloblastomas depend on *Eed* and the PRC2, we bred *Eed*^*cKO*^ and *Ezh2*^*cKO*^ mice with *SmoM2* mice [52]. *SmoM2* mice harbor a *Cre*-conditional transgene with an oncogenic allele of the SHH receptor component *Smo*. Mice that inherit both *Math1-Cre* and *SmoM2* develop medulloblastoma with 100% penetrance and without treatment die of tumor progression by P50 [6]. We have shown that these tumors recapitulate the gene expression patterns and cellular diversity of SHH medulloblastomas resected from patients [3, 38, 53]. By interbreeding *Eed*^*cKO*^ and *Ezh2*^*cKO*^ mice with *SmoM2* mice, we generated pups with the genotypes *Math1-Cre/Eed*^*fl/fl*^*/SmoM2* (*M-Smo/Eed*^*cKO*^) and *Math1-Cre/Ezh2*^*fl/fl*^*/SmoM2* (*M-Smo/Ezh2*^*cKO*^). To generate control mice with SHH medulloblastomas with intact PRC2, we bred *Math1-Cre* and *SmoM2* mice to generate *Math1-Cre/SmoM2* (*M-Smo*) controls. We then compared medulloblastomas in *M-Smo/Eed*^*cKO*^, *M-Smo/Ezh2*^*cKO*^, and *M-Smo* mice.

**Fig. 5:**
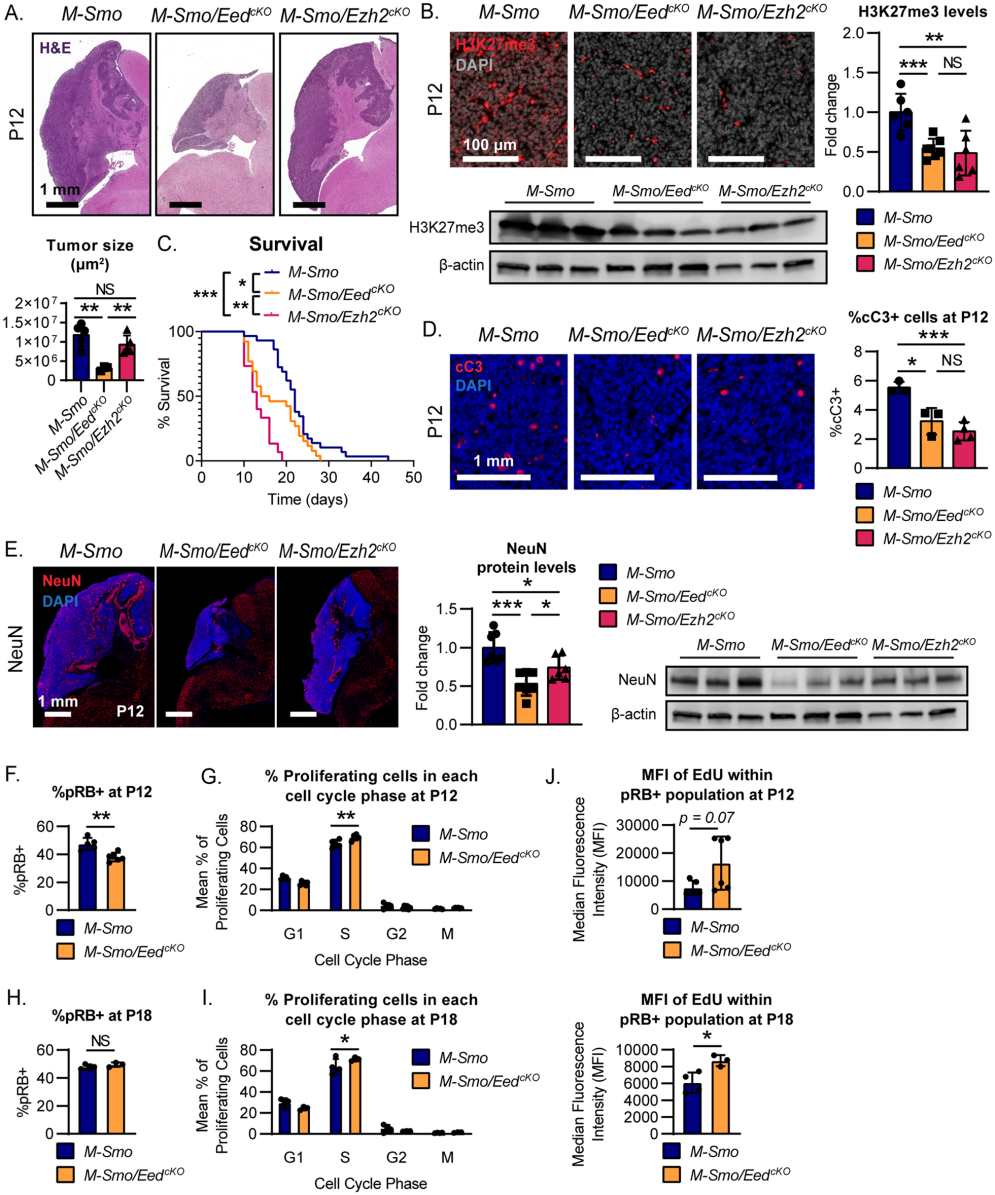
Loss of PCR2 function accelerated progression of SHH medulloblastomas. **A** Representative H&E-stained sagittal sections of medulloblastomas in *M-Smo*, *M-Smo/Eed*^*cKO*^, and *M-Smo/Ezh2*^*cKO*^ mice at postnatal day 12 (P12) and comparison of cross-sectional area of tumors in midline sections of replicate mice of each genotype, usiong two-tailed Student’s *t*-test. **B** Representative IF stains showing H3K27me3 in *M-Smo*, *M-Smo/Eed*^*cKO*^, and *MSmo/Ezh2*^*cKO*^ tumors, and H3K27me3 western blots of medulloblastomas from 3 replicate mice of each genotype. **C** Kaplan–Meier curves compare the survival times of *M-Smo*, *M-Smo/Eed*^*cKO*^, and *MSmo/Ezh2*^*cKO*^ mice, using the Log-Rank test. **D** IHC for cC3 in representative sections, with quantitative analysis of replicate samples, compared by two-tailed Student’s *t*-test. **E** IHC for NEUN in representative sections, with western blot of replicate samples quantified on the right, compared by two-tailed Student’s *t*-test. **F–J** Flow cytometry analysis of dissociated tumors of indicated age and genotype, showing **F, H** pRB + fractions and **G, I** cell cycle distribution of pRB + cells, and **J** EdU MFI, compared by two-tailed Student’s *t*-test. *, **, and *** denote *p* < 0.05, *p* < 0.01 and *p* < 0.001 respectively, relative to controls

*M-Smo/Eed*^*cKO*^*, M-Smo/Ezh2*^*cKO*^, and *M-Smo* mice all developed medulloblastomas with 100% frequency by P10 (Fig. 5A). *M-Smo* tumors showed heterogeneous H3K27me3, with strongest H3K27me3 expression in the most differentiated elements, similar to WT cerebella (Fig. 5B). Deletion of either *Eed* or *Ezh2* disrupted PRC2 function, as both *M-Smo/Eed*^*cKO*^ and *M-Smo/Ezh2*^*cKO*^ showed minimal H3K27me3 in tumor cells, with residual H3K27me3 in interspersed stromal cells demonstrating the effectiveness of the staining technique (Fig. 5B). *M-Smo/Eed*^*cKO*^ and *M-Smo/Ezh2*^*cKO*^ mice showed shorter survival times compared to controls, indicating that PRC2-mutant medulloblastomas progressed more quickly (Fig. 5C).

We compared apoptosis and terminal differentiation in *M-Smo/Eed*^*cKO*^, *M-Smo/Ezh2*^*cKO*^, and *M-Smo* tumors. *Eed*-deleted and *Ezh2*-deleted medulloblastomas showed less apoptosis, demonstrated smaller fractions of cC3 + cells compared to control tumors (Fig. 5D), in contrast to the increased cell death that we noted in *Eed*^*cKO*^ cerebella. Medulloblastomas in *M-Smo/Eed*^*cKO*^ and *M-Smo/Ezh2*^*cKO*^ mice also showed reduced neuronal differentiation, demonstrated by less abundance of neuronal marker NEUN (Fig. 5E). PRC2 disruption therefore decreased both apoptosis and terminal differentiation in SHH medulloblastoma.

### Transient growth suppression in ***M-Smo/Eed***^***cKO***^ medulloblastomas

While *M-Smo/Eed*^*cKO*^ tumors progressed more rapidly than *M-Smo* control tumors, we noted consistently smaller cross-sectional area in midline sections of *M-Smo/Eed*^*cKO*^ tumors at P12 compared to either *M-Smo/Ezh2*^*cKO*^ or control tumors (Fig. 5A), suggesting that *Eed* deletion might produce an initial growth suppression, followed by more rapid growth. To analyze tumor growth dynamics, we compared RB phosphorylation and cell cycle progression in *M-Smo/Eed*^*cKO*^ and *M-Smo* tumors. We injected EdU into 3–5 replicate mice of each genotype at either P12 or P18, then harvested tumors 1 h after EdU injection and quantified pRB and EdU uptake by flow cytometry.

P12 *M-Smo/Eed*^*cKO*^ tumors showed smaller fractions of pRB + cells compared to P12 *M-Smo* control tumors (Fig. 5F), indicating that fewer tumor cells were proliferative. Within the pRB + fractions of P12 *M-Smo/Eed*^*cKO*^ tumors, however, more cells were in S-phase compared to the pRB + fractions of P12 *M-Smo* tumors (Fig. 5G), indicating more rapid progression from G_1_. P12 *M-Smo/Eed*^*cKO*^ tumors thus contained smaller proliferative populations, consistent with smaller tumor size. However, the cells that were proliferating in P12 *M-Smo/Eed*^*cKO*^ medulloblastomas were cycling more rapidly, suggesting a transition to faster tumor growth.

Consistent with more rapid tumor growth after P12, by P18 *M-Smo/Eed*^*cKO*^ tumors no longer showed fewer pRB + cells (Fig. 5H). Moreover, pRB + cells in *M-Smo/Eed*^*cKO*^ tumors continued to show more rapid cycling, demonstrated by greater S-phase fractions, compared to pRB + cells from P18 *M-Smo* tumors (Fig. 5I). Supporting the more rapid S-phase progression in *M-Smo/Eed*^*cKO*^ tumors at both P12 and P18, pRB + cells from *M-Smo/Eed*^*cKO*^ tumors showed higher EdU median fluorescence intensity (MFI), indicating increased EdU uptake within the period of EdU exposure (Fig. 5J). The smaller pRB + population in *M-Smo/Eed*^*cKO*^ tumors at P12, the increased rate of proliferation within the pRB + population, and the similar pRB + population at P18 are all consistent with a biphasic effect of *Eed* deletion on tumor growth, in which tumors initially grew more slowly and then accelerated, producing shorter survival times.

### Up-regulation of PRC2 target genes without growth suppression in PRC2-mutant medulloblastomas

**Fig. 6:**
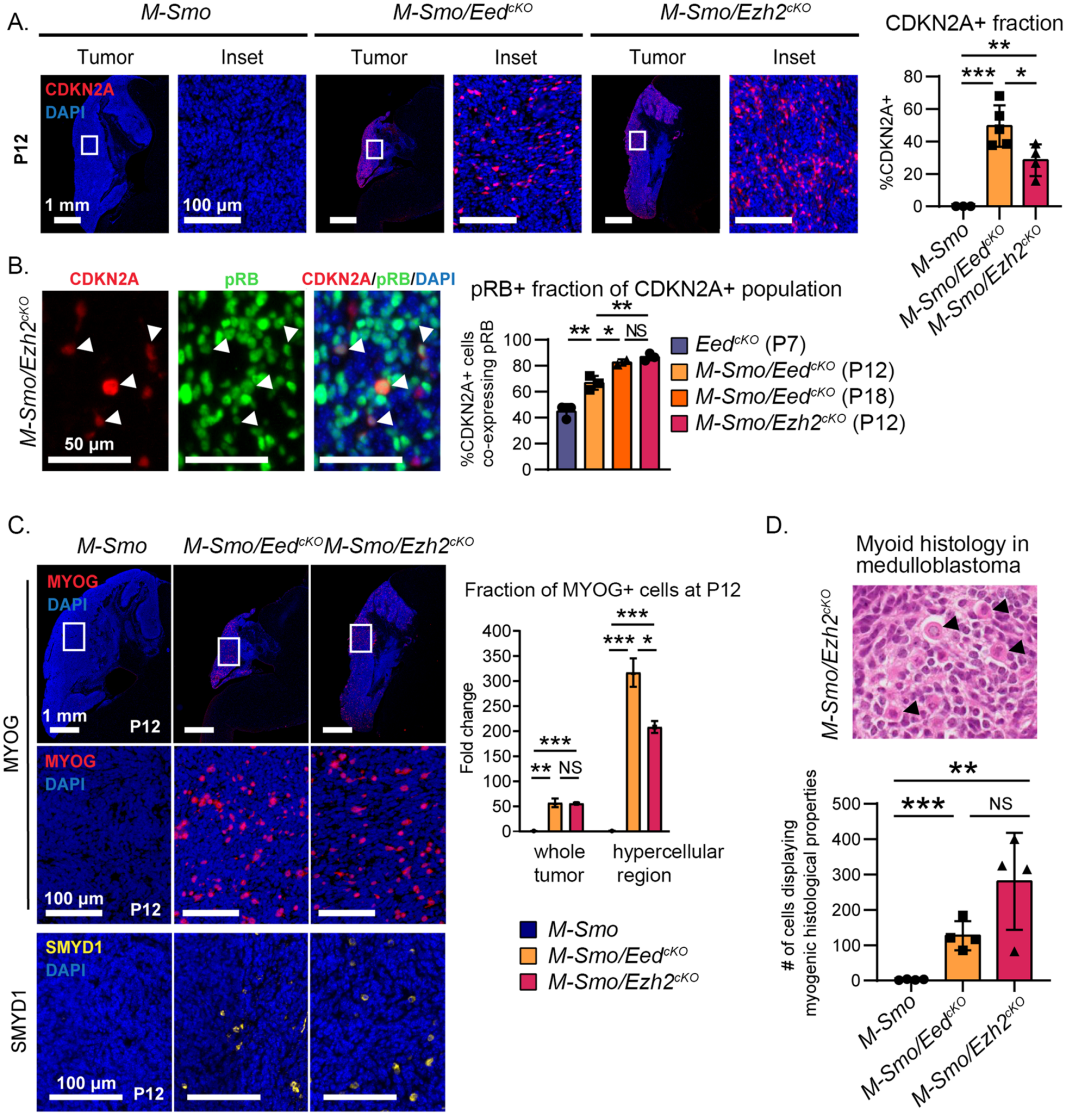
Up-regulation of PRC2 targets and myoid differentiation in both *Eed*-deleted and *Ezh2-*deleted SHH medulloblastomas. **A** Representative CDKN2A IHC in medulloblastomas of indicated genotypes, with quantification in replicate mice of each genotype. **B** Representative images of CDKN2A/pRB dual staining and quantification of pRB + fractions of CDKN2A-expressing cells in the indicated ages and genotypes. **C** Representative MYOG and SMYD1 IHC in medulloblastomas of indicated genotypes with quantification of MYOG + cells in replicate mice of each genotype. **D** Representative H&E-stained section from a medulloblastoma in a *M-Smo/Ezh2*^*cKO*^ mouse, with myoid cells (arrows), and quantification in replicate mice of the indicated genotypes. *, **, and *** denote *p* < 0.05, *p* < 0.01 and *p* < 0.001 respectively, relative to controls

We investigated whether medulloblastomas with deletion of *Eed* or *Ezh2* up-regulated the same PRC2 targets that were up-regulated in *Eed*^*cKO*^ CGNPs. Both *M-Smo/Eed*^*cKO*^ and *M-Smo/Ezh2*^*cKO*^ medulloblastomas showed frequent CDKN2A+ cells which were not observed in *M-Smo* control tumors (Fig. 6A). CDKN2A suppressed RB phosphorylation less effectively in tumor cells than in CGNPs, as the pRB+ fractions of CDKN2A+ cells were significantly higher in *M-Smo/Eed*^*cKO*^ and *M-Smo/Ezh2*^*cKO*^ medulloblastomas compared to P7 *Eed*^*cKO*^ cerebella (Fig. 6B). Moreover, the pRB+ fraction of CDKN2A+ cells correlated with tumor growth; *M-Smo/Ezh2*^*cKO*^ tumors, which progressed faster than *M-Smo/Eed*^*cKO*^ tumors, showed higher pRB+ fractions of CDKN2A+ cells at P12. By P18, when proliferation accelerated in *M-Smo/Eed*^*cKO*^ tumors, the pRB+ fractions of CDKN2A+ cells was also increased, to become similar to the P12 *M-Smo/Ezh2*^*cKO*^ tumors. CDKN2A was thus up-regulated in both *Eed*-deleted and *Ezh2*-deleted medulloblastomas but did not restrict RB phosphorylation or tumor progression.

Analysis of muscle markers MYOG and SMYD1 showed inappropriate myoid differentiation in *M-Smo/Eed*^*cKO*^ and *M-Smo/Ezh2*^*cKO*^ tumors (Fig. 6C), as seen in *Eed*^*cKO*^ cerebella. The expression of muscle genes in *M-Smo/Eed*^*cKO*^ and *M-Smo/Ezh2*^*cKO*^ medulloblastomas suggested similarity to the clinically observed medulloblastoma variant, medullomyoblastoma. To determine if the *M-Smo/Eed*^*cKO*^ and *M-Smo/Ezh2*^*cKO*^ tumors recapitulated medullomyoblastoma histopathology, we submitted H&E sections from replicate *M-Smo/Eed*^*cKO*^*, M-Smo/Ezh2*^*cKO*^, and *M-Smo* tumors to a blinded analysis. Two experienced pediatric neuropathologists counted myoid cells, defined by key morphologic changes (Fig. 6D), in replicate sections, while blinded to the genotype. The resulting quantifications of myoid cells correctly distinguished control tumors from tumors with either *Eed* or *Ezh2* deletion, which were not significantly different from each other (Fig. 6E). Deletion of either *Eed* or *Ezh2* was therefore sufficient to allow myoid differentiation, reproducing the molecular and histologic features of medullomyoblastoma.

## Discussion

Our data show that the PRC2 maintains neuronal fate commitment in cerebellar progenitors and in SHH medulloblastoma by preventing alternative, myoid differentiation. In the postnatal cerebellum, conditional deletion of PRC2 components *Ezh2* and *Eed* in the *Atoh1* lineage disrupted PRC2-mediated H3K27 trimethylation and caused a fraction of CGNPs to differentiate along a muscle cell trajectory. *Eed* deletion induced myoid differentiation in more cells than *Ezh2* deletion and markedly impaired cerebellar growth through a combination of decreased proliferation and increased apoptosis. In medulloblastomas, deletion of either *Eed* or *Ezh2* resulted in myoid differentiation, but neither deletion increased apoptosis or durably prevented tumor growth. Rather, deletion of either *Eed* or *Ezh2* accelerated tumor progression.

Single-cell transcriptomic analysis of postnatal cerebella showed that *Eed*-deleted CGNPs inappropriately expressed genes typically suppressed by the PRC2, including *Cdkn2a, Hoxa9*, and *Hoxa7*. Up-regulation of *Cdkn2a* may be sufficient to explain reduced CGNP proliferation, as seen in *Eed*-deleted hippocampal progenitors [11]. Up-regulation of specific BH3-only genes suggested that direct activation of BAX or BAK may mediate increased CGNP apoptosis. Co-deletion studies of *Eed* plus either *Trp53* or *Bax* AND *Bak* confirmed that increased apoptosis occurred by p53-independent activation of the intrinsic apoptosis pathway. The partial rescue of cerebellar growth in *Eed/Bax/Bak*^*tKO*^ mice demonstrates that inappropriate apoptosis contributed to growth failure and restricted the myoid population.

PRC2 function was not required for neural differentiation, as in *Eed*-deleted cerebella, most CGNPs were able to complete neural differentiation, achieving the neural fates of Clusters 4_1 and 8 in *Eed*^*cKO*^ mice. The inappropriate expression of *Hox* genes in these clusters did not prevent a recognizable CGN-like pattern of gene expression. In contrast, the myoid differentiation of Cluster 15 demonstrates that PRC2 disruption permitted new fate possibilities.

The specific diversion of CGNPs and medulloblastoma cells into myoid fates may be related to the normal expression of the MYOD1 transcription factor during postnatal development. MYOD1 is an early myogenic transcription factor that activates MYOG in developing muscle progenitors. Prior studies show that WT P7 cerebella and SHH medulloblastomas contain populations of cells that express MYOD1 without inducing MYOG or activating a myogenic program [3, 54]. In the *Eed*-deleted and *Ezh2*-deleted cerebella and medulloblastomas, however, *Myod1*+ cells also expressed *Myog* and adopted a myogenic trajectory. These data suggest that one function of the PRC2 in CGNPs is to suppress *Myog* and other myoid genes, allowing CGNPs to use the MYOD1 transcription factor to regulate neural development, without risk of inappropriate differentiation. We propose more generally that by suppressing inappropriate differentiation pathways, the PRC2 allows specifically neural functions of transcriptional regulators such as MYOD1, that have non-neural functions in other types of cells.

*Eed* deletion resulted in cerebellar hypoplasia that was not seen in *Ezh2*-deleted mice. The difference in phenotype may result from a more severe PRC2 disruption caused by loss of EED. In other cell types, EED protein is required for the stability of the other components of the PRC2 [30] and for PRC2 methyltransferase activity [55]. Consistent with these prior reports, we found that *Eed*^*cKO*^ CGNPs lacked both EED and EZH2 proteins, indicating destabilization of the entire PRC2. In contrast, EZH2 is not required for PRC2 stability and can be partially compensated by the homolog EZH1 in multiple cellular contexts [56, 57]. The PRC2 components that persist in *Ezh2*-deleted CGNPs, possibly with compensation from EZH1, may retain sufficient function to sustain cerebellar growth, and to suppress PRC2 target gene expression in most but not all CGNPs, resulting in fewer myoid cells in *Ezh2*^*cKO*^ cerebella compared to *Eed*^*cKO*^ cerebella. Different phenotypes were similarly noted when either *Eed* or *Ezh2* were deleted in intestinal epithelia [44]. Conditional deletion of *Eed* in the intestinal crypts decreased proliferation and caused hypoplasia, while *Ezh2* deletion did not cause an overt phenotype. The continued proliferation in *Ezh2*-deleted intestinal crypts suggests that EZH1 may compensate for EZH2 loss in these cells [44], and a similar mechanism may explain sustained proliferation of *Ezh2-*deleted CGNPs.

Alternatively, as a shared component of the PRC1 and PRC2 complexes, EED loss may more broadly affect chromatin repression [58]. Our finding that *Eed* deletion did not affect levels of H2AK119 monoubiquitylation suggests that PRC1 activity was not altered in *Eed*^*cKO*^ CGNPs or medulloblastomas. The overlapping patterns of differential gene expression in *Eed*^*cKO*^ and *Ezh2*^*cKO*^ cerebella show that suppression of myoid differentiation in CGNPs depends on PRC2 function. We cannot, however, exclude the possibility that the growth-suppressive effects of *Eed* deletion are mediated by functions of EED protein outside of the PRC2.

In SHH medulloblastomas, disrupting PRC2 activity through deletion of either *Eed* or *Ezh2* was sufficient to allow widespread expression of genes typically suppressed by the PRC2, including the CDKN2A tumor suppressor. Neither PRC2 disruption nor CDKN2A expression, however, was sufficient for sustained suppression of tumor growth. *Eed* deletion reduced the overall proliferation rate in each P12 *M-Smo/Eed*^*cKO*^ tumor, producing transient growth suppression. Over time, however, a fraction of *Eed*-deleted tumor cells that were rapidly proliferative increased, driving ultimately faster progression. The initial reduction in tumor growth in *M-Smo/Eed*^*cKO*^ tumors was consistent with previous studies that showed anti-tumor effects of PRC2 disruption using EZH2 inhibitor treatment in models SHH medulloblastoma [22, 59, 60]. However, the shorter survival times in *M-Smo/Eed*^*cKO*^ and *M-Smo/Ezh2*^*cKO*^ mice raise concern that PRC2 disruption may not produce durable anti-tumor effects.

Our genetic studies identify a role of PRC2 in regulating neural fate commitment of cerebellar progenitors and medulloblastoma cells, and implicate PRC2 disruption in the pathogenesis of medullomyoblastoma, a subtype of medulloblastomas characterized by myogenic differentiation of tumor cells [61, 62]. Our data also caution that pharmacologically inhibiting PRC2 function in medulloblastoma may hasten, rather than slow, tumor growth.

## Methods and materials

### Mice

We generated *Ezh2*^*cKO*^ mice by breeding *Math1-Cre* mice (Jackson Labs Stock #011104), which express Cre recombinase in CGNPs, with *Ezh2*^*LoxP/LoxP*^ (Jackson Labs Stock #022616). We generated *Eed*^*cKO*^ mice by breeding *Math1-Cre* mice with *Eed*^*LoxP/LoxP*^ mice (generously donated by Dr. Terry Magnuson). To generate *Eed/Tp53*^*dKO*^ mice, we interbred *Math1-Cre/Eed*^*LoxP/LoxP*^ and *Trp53*^*LoxP/LoxP*^ mice (Jackson Labs stock # 008462). To generate *Eed/Bax/Bak*^*tKO*^ mice, we interbred *Math1-Cre/Eed*^*LoxP/LoxP*^ and *Bax*^*LoxP/LoxP*^*/Bak*^*−/−*^ mice (Jackson Labs stock # 006329).

To generate *M-Smo* mice, we crossed *Math1-Cre* mice with *SmoM2* mice (Jackson Labs stock #005131) that harbor a Cre-conditional transgene comprising of an oncogenic allele of *Smo*, fused to the YFP coding sequence. We then crossed *Math1-Cre/Ezh2*^*LoxP/LoxP*^ and *Ezh2*^*LoxP/LoxP*^*/SmoM2*^*LoxP/LoxP*^ mice to produce *M-Smo/Ezh2*^*cKO*^ mice and *Math1-Cre/Eed*^*LoxP/LoxP*^ and *Eed*^*LoxP/LoxP*^*/SmoM2*^*LoxP/LoxP*^ mice to produce *M-Smo/Eed*^*cKO*^ mice.

All mice were of species *Mus musculus* and crossed into the C57BL/6 background through at least five generations.

### Histology and immunohistochemistry

Mouse brains were processed, immunostained, and quantified as previously described [67–69]. In brief, mice were placed under isoflurane anesthesia and decapitated. Harvested brains were fixed by immersion in 4% formaldehyde for 24 h and then transferred to a graded ethanol series and embedded in paraffin and sectioned along the sagittal midline. Samples were stained and imaged using an Aperio Scanscope and quantified via automated cell counting using Tissue Studio (Definiens).

Primary antibodies used were: H3K27me3 diluted 1:200 (Cell Signaling, #9733), pRB diluted 1:3000 (Cell Signaling, #8516), cC3 diluted 1:400 (Biocare Medical, #CP229C), NeuN diluted 1:10,000 (Millipore, MAB377), Myogenin (MYOG) diluted 1:500 (Abcam, ab124800), SMYD1 diluted 1:100 (ThermoFisher, PA5-84544), and CDKN2A diluted 1:500 (Abcam, ab241543). Stained images were counterstained with DAPI.

### Western blot

Whole cerebella or tumors were harvested and homogenized in an SDS lysis buffer, which included SDS Solution (20%) (ThermoFisher 151-21-3), UltraPure™ 1 M Tris-HCI Buffer, pH 7.5 (ThermoFisher 15567027), UltraPure™ 0.5 M EDTA, pH 8.0 (ThermoFisher 15575038), Phenylmethanesulfonyl fluoride (≥ 98.5%; powder) (Millipore Sigma 329-98-6), Isopropyl alcohol (Mallinckrodt 3037), and purified water. Equal total protein concentrations were loaded from each sample and run on SDS-polyacrylamide gels (BioRad, #4561105, #4568094), transferred onto polyvinylidene difluoride membranes, and membranes blotted using a SNAP i.d. 2.0 Protein Detection System (Millipore). The following antibodies were used: H3K27me3 (Cell Signaling, #9733; 1:500 dilution), H3K4me3 (Cell Signaling, #9751; 1:500 dilution), H2AK119ub (Cell Signaling, #8240; 1:500 dilution), H3K27Ac (Cell Signaling, #8173; 1:500 dilution), NeuN (Millipore, MAB377; 1:500 dilution), β-actin (Cell Signaling, #3700; 1:5000 dilution), Anti-rabbit IgG, HRP-linked antibody (Cell Signaling, #7074; 1.5:1000 dilution), and Anti-mouse IgG, HRP-linked antibody (Cell Signaling, #7076; 1.5:1000 dilution). Membranes were imaged using a chemiluminescent SuperSignal West Femto Maximum Sensitivity Substrate (34095, Thermo Fisher Scientific) and the C-DiGit blot scanner (LI-COR Biosciences). Blots were then quantified using Image Studio Lite software (LI-COR).

### Flow cytometry: cell cycle analysis

P12 and P18 *M-Smo* and *M-Smo/Eed*^*cKO*^ mice were injected with Edu 1 h prior to harvest. Mice were anesthetized with isoflurane and decapitated. Tumors samples were dissociated using the Cell Dissociation Kit (Worthington Biochemical Corporation, #LK003150), which included dissociation with papain at 37 °C for 15 min, and isolation using an ovomucoid inhibitor density gradient. Tumor cells were then treated with the Fixation and Permeabilization Kit (Life Technologies, #GAS004), and stained using the following antibodies: 647-conjugated pRB diluted 1:50 (Cell Signaling, #8974) and FxCycle Violet at 1:100 (Life Technologies, #F10347). Edu was detected using a Click-iT EdU Alexa Fluor 488 Imaging Kit (catalogue number C10337; Life Sciences). Samples were run on an LSRFortessa (BD Biosciences) at the UNC Flow Cytometry Core. Data was analyzed using Flow Jo v10.

### Single cell sequencing (scRNA-seq): sample collection

Brains were harvested and cut along the sagittal midline. One half of the cerebellum from each mouse was dissociated and processed for scRNA-seq, and the other half of the brain was fixed, sectioned, and analyzed to confirm phenotype. Half cerebella were processed using the Cell Dissociation Kit (Worthington Biochemical Corporation, #LK003150), in which samples were treated with papain at 37 °C for 15 min and then separated by centrifugation of an ovomucoid inhibitor density gradient. Cells were then subjected to bead pairing by microfluidics, cDNA synthesis, and library construction using the Drop-seq V3 method [66] as in our prior studies [70–73].

### scRNA-seq: processing data

Data analysis was performed using the Seurat R package version 3.1.1 [74]. Data were subjected to several filtering steps. Genes detected in > 30 cells were filtered out, to prevent misaligned reads appearing as rare transcripts in the data. Putative cells with fewer than 500 detected RNA molecules (nCount) or 200 different genes (nFeature) were considered to have too little information to be useful, and potentially to contain mostly ambient mRNA reads. Putative cells with greater than 4 standard deviations above the median nCount or nFeature were suspected to be doublets, improperly merged barcodes, or sequencing artifacts, and were excluded. As in our previously published work, putative cells with more than 10% mitochondrial transcripts were suspected to be dying cells and also excluded [70].

In total, 86% of putative cells from WT mice and 74% of putative cells from *Eed*^*cKO*^ mice met QC criteria and were included in the analysis. From the 5 WT mice, we included a total of 6558 cells with a range of 673–1852 cells per animal and a median of 1138 cells. From the 3 *Eed*^*cKO*^ mice, we included a total of 2576 cells, with a range of 692–1036 cells per animal and a median of 847 cells.

### scRNA-seq: data normalization, clustering, differential gene expression, and cell type identification

The data was normalized using the SCTransform method as implemented in Seurat. The function then selected the top 3000 most highly variable genes. PCA was performed on the subset of highly variable genes using the RunPCA function. We used 15 PCs in downstream analysis, based on examining the elbow in the elbow plot as implemented by Seurat. We identified cell clusters using the FindNeighbors and FindClusters functions.

To identify differential genes between clusters of cells, we used the Wilcoxon rank sum test to compare gene expression of cells within the cluster of interest to all cells outside that cluster, implemented by the FindMarkers function. Uniform Manifold Approximation and Projection (UMAP) was used to reduce the PCs to two dimensions for data visualization using the RunUMAP function. For re-iterated analysis of the Clusters 4 and 6, the same procedures were used. We then determined the type of cell within each cluster by analyzing cluster-specific gene expression patterns.

### Pathology scoring

Sagittal H&E sections of P12 *M-Smo*, *M-Smo/Eed*^*cKO*^, and *M-Smo/Ezh2*^*cKO*^ mouse brains were analyzed by neuropathologists (MS and JV) while blinded to the genotype, and the number of myoid cells per sample were manually counted.

### Survival curves

Tumor-bearing mice were monitored daily and harvested according to a pre-determined humane endpoint, which included a decrease of weight > 10% overnight, a hunched posture, decreased mobility or inability to eat, and ataxia.

### Statistical analyses

Two-tailed Student’s t-tests were used to compare IHC and western blot quantifications between *Ezh2*^*cKO*^ and *Eed*^*cKO*^ genotypes. One-tailed Student’s *t*-tests were used to compare these genotypes versus controls for markers that were absent in controls. The Barnard’s exact test was used to make comparisons between categorical variables in comparisons of markers that were determined to be present or absent in individual replicates. Survival curves were compared using the Log-rank (Mantel-Cox) test. Dirichelet regression analysis was performed in R using the DirchletReg 0.7-1 package [63].

## Supplementary Information

**Additional file 1: Figure S1**. Initial uniform manifold approximation and projection (UMAP) qualitative map of cells dissociated from harvested cerebella from 5 WT and 3 *Eed*^*cKO*^ mice. Cells were subdivided into 20 color-coded clusters. **Figure S2** Disabling apoptosis increased the myoid population. Representative images of MYOG IHC in cerebella of indicated genotypes show that a population of myoid cells persisted in *Eed*^*cKO*^ cerebella at P21, and that the myoid population increased when apoptosis was blocked by deletion of both *Bax* AND *Bak*. The increased myoid cells in *Eed/Bax/Bak*^*tKO*^ cerebella indicates that apoptosis decreases the myoid population in *Eed*^*cKO*^ cerebella

**Additional file 2**. Cluster-specific sets of differentially expressed genes, with statistical analyses.

## Data Availability

The scRNA-seq data are publicly available in the GEO database under accession number GSE129730 for controls and GSE207451 for *Eed*^*cKO*^ cerebella.
