# Supplementary material for: PRC2 disruption in cerebellar progenitors produces cerebellar hypoplasia and aberrant myoid differentiation without blocking medulloblastoma growth
Source: Acta Neuropathol Commun. 2023 Jan 12;11:8. doi: 10.1186/s40478-023-01508-x (PMC9838053; doi:10.1186/s40478-023-01508-x)
Supplement: Supplementary file 1 — Additional file 1: Figure S1. Initial uniform manifold approximation and projection (UMAP) qualitative map of cells dissociated from harvested cerebella from 5 WT and 3 EedcKO mice. Cells were subdivided into 20 color-coded clusters. Figure S2 Disabling apoptosis increased the myoid population. Representative images of MYOG IHC in cerebella of indicated genotypes show that a population of myoid cells persisted in EedcKO cerebella at P21, and that the myoid population increased when apoptosis was blocked by deletion of both Bax AND Bak. The increased myoid cells in Eed/Bax/BaktKO cerebella indicates that apoptosis decreases the myoid population in EedcKO cerebella [file 40478_2023_1508_MOESM1_ESM.docx]

**Supplementary Figures**

**
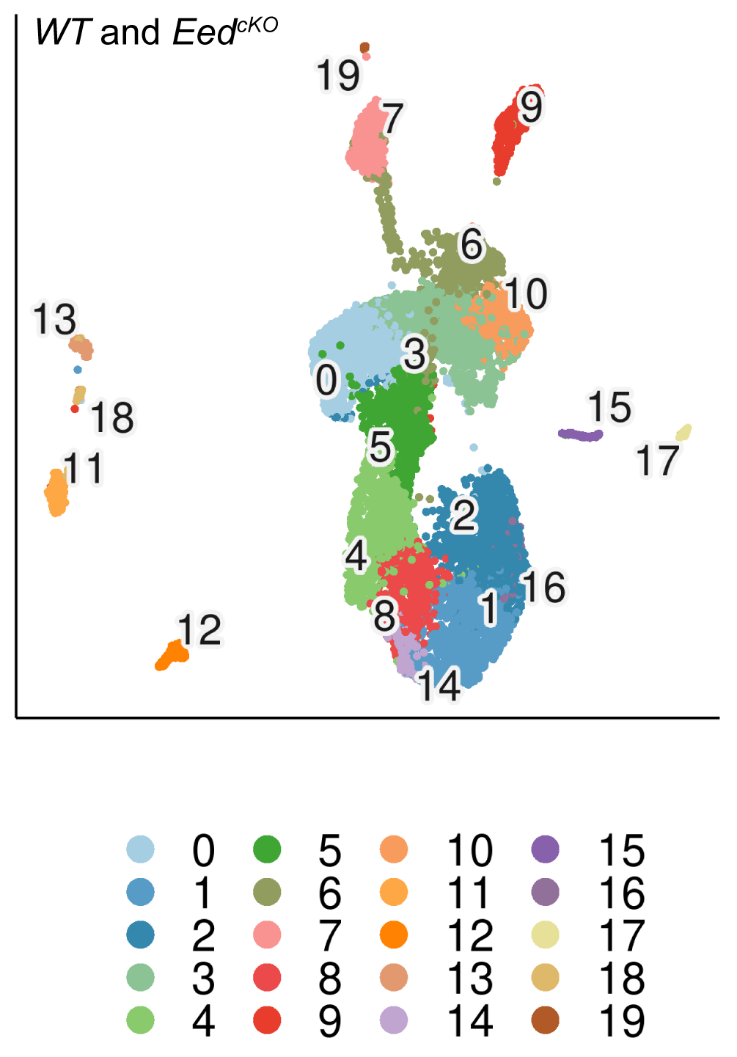
**

**Supplementary Figure 1.** Initial uniform manifold approximation and projection (UMAP) qualitative map of cells dissociated from harvested cerebella from 5 WT and 3 *Eed^cKO^* mice. Cells were subdivided into 20 color-coded clusters.


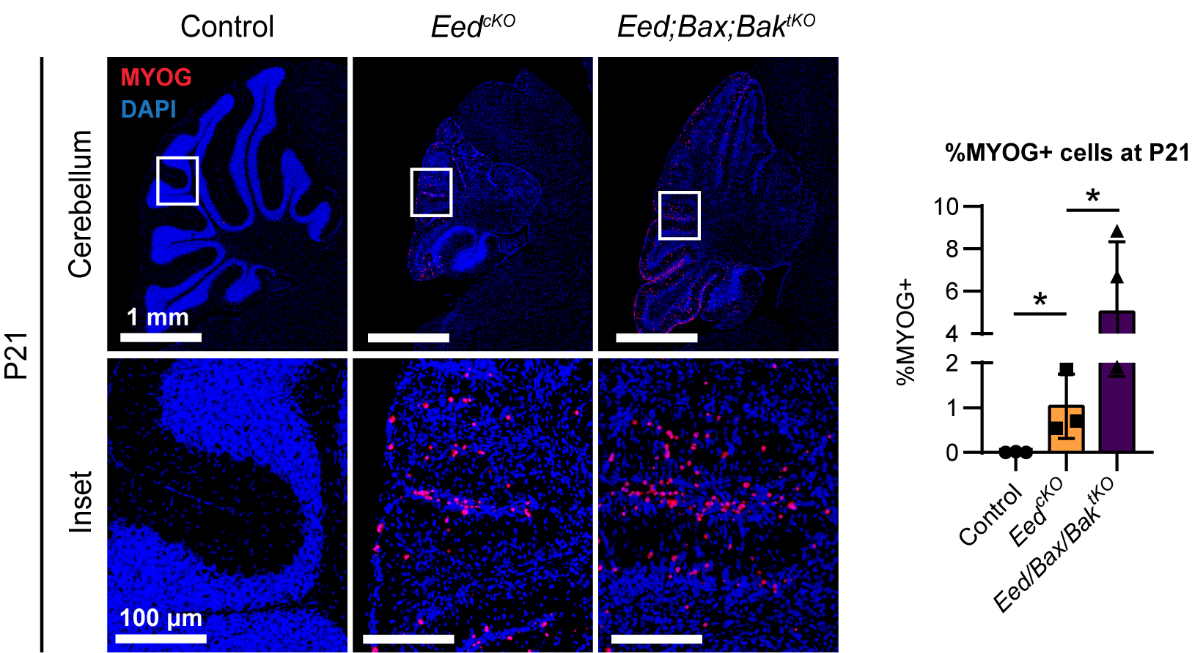


**Supplemental Fig. 2: Disabling apoptosis increased the myoid population.** Representative images of MYOG IHC in cerebella of indicated genotypes show that a population of myoid cells persisted in *Eed^cKO^* cerebella at P21, and that the myoid population increased when apoptosis was blocked by deletion of both *Bax* AND *Bak*. The increased myoid cells in *Eed/Bax/Bak^tKO^* cerebella indicates that apoptosis decreases the myoid population in *Eed^cKO^* cerebella.
